# GLP-1 and Parkinson’s Disease: A Comprehensive Review of Biology, Mechanisms and Efficacy

**DOI:** 10.3390/cells15090804

**Published:** 2026-04-29

**Authors:** Roxana Mezabrovschi, Matthew E. Gegg, Anthony H. V. Schapira

**Affiliations:** Department of Clinical and Movement Neurosciences, UCL Queen Square Institute of Neurology, London WC1N 3BG, UK; r.mezabrovschi@ucl.ac.uk (R.M.); matthew.gegg@ucl.ac.uk (M.E.G.)

**Keywords:** glucagon-like peptide-1, GLP-1 receptor, GLP-1 receptor agonists, Parkinson’s disease, gut–brain axis, neuroprotection, GPCR signalling, neuroinflammation

## Abstract

**Highlights:**

**What are the main findings?**

- GLP-1 receptor (GLP-1R) signalling integrates metabolic and neurodegenerative biology, engaging conserved intracellular pathways, including cAMP/PKA, PI3K/Akt, autophagy-lysosome regulation, mitochondrial homeostasis, and inflammatory control, which are all disrupted in Parkinson’s disease (PD).
- Experimental and early clinical evidence indicates that GLP-1 receptor agonists (GLP-1RAs) engage disease-relevant central nervous system (CNS) pathways, modulating dopaminergic neuron vulnerability, glial reactivity, and cellular stress responses across experimental models and selected patient cohorts.

**What are the implications of the main finding?**

- GLP-1R signalling constitutes a biologically plausible therapeutic target in PD.
- Clarifying CNS target engagement, peripheral-CNS communication, and disease-stage specificity will be essential to determine the translational relevance of GLP-1RAs for PD.

**Abstract:**

Neurodegenerative disorders, including Parkinson’s disease (PD), are largely treated with symptomatic therapies, underscoring the need for strategies that target underlying disease mechanisms. Glucagon-like peptide-1 (GLP-1) and its receptor (GLP-1R), a class B G protein-coupled receptor best known for metabolic regulation, have attracted interest due to the increasing evidence of central nervous system (CNS) actions. This review synthesises mechanistic, preclinical, and clinical evidence examining GLP-1R signalling in PD and related neurodegenerative contexts. We integrate findings from cellular and animal models with early-phase clinical studies of GLP-1 receptor agonists (GLP-1RAs). Across experimental systems, GLP-1R activation engages conserved intracellular pathways—cAMP/PKA, PI3K/Akt, and ERK—that regulate mitochondrial function, oxidative stress, autophagy-lysosomal dynamics, and inflammatory signalling. In PD-relevant models, these pathways intersect with key pathogenic features, including α-synuclein accumulation, dopaminergic neuron vulnerability, and glial reactivity. Clinical studies to date demonstrate acceptable safety and tolerability, alongside biomarker evidence of central pathway engagement and variable effects on motor and non-motor outcomes. However, uncertainties remain regarding CNS target engagement, peripheral versus CNS mechanisms, and disease-stage dependence. Overall, the current evidence positions GLP-1R signalling as a biologically plausible therapeutic pathway in PD that warrants further mechanistic clarification and rigorous evaluation in ongoing and future clinical trials.

## 1. Introduction

Neurodegenerative disorders, including Alzheimer’s disease (AD) and Parkinson’s disease (PD), are characterised by progressive neuronal dysfunction and loss. These changes lead to cognitive decline, motor impairment, and a broad range of other symptoms. Together, these conditions represent a growing global health burden driven by ageing populations and the limited availability of therapies that meaningfully alter disease progression. Current clinical interventions remain largely symptomatic and do not halt or reverse disease progression, underscoring the urgent need to identify novel therapeutic strategies that target the fundamental mechanisms of neurodegeneration.

Increasing attention has therefore turned to systemic and metabolic pathways that may influence neurodegenerative processes through coordinated central nervous system (CNS) and peripheral mechanisms. Among these, glucagon-like peptide-1 (GLP-1) and its receptor (GLP-1R) have emerged as candidates of particular interest. Although GLP-1 was initially characterised for its role in glucose metabolism, accumulating experimental evidence indicates that GLP-1 signalling influences multiple CNS-relevant processes, including neuronal survival, synaptic function, and neuroinflammation [1,2,3,4,5]. This is particularly relevant to PD, where epidemiological and mechanistic studies increasingly support links between T2DM, insulin resistance, and neurodegenerative vulnerability. However, these associations are not uniform across studies and are likely influenced by multiple confounding factors, including vascular comorbidity, medication exposure, and differences in study design.

GLP-1 is an endogenous 30-amino acid peptide hormone produced in intestinal endocrine L-cells [6]. Its biological effects are mediated through the GLP-1R, a class B G protein-coupled receptor (GPCR). The receptor consists of a large extracellular domain that binds the peptide’s C-terminal segment and a seven-transmembrane helical domain that engages the N-terminal region [7]. This structural organisation supports ligand-receptor coupling and downstream signal transduction, which underpins GLP-1’s classical metabolic effects and provides a mechanistic basis for its emerging actions within the CNS. As such, GLP-1R signalling is increasingly investigated as a mechanistic interface linking metabolic regulation, gut–brain communication, and neurodegenerative disease pathways.

Physiologically, GLP-1 regulates glucose homeostasis by stimulating glucose-dependent insulin secretion, suppressing glucagon release, delaying gastric emptying, and reducing appetite [6]. The clinical development of GLP-1 receptor agonists (GLP-1RAs) for the treatment of type 2 diabetes mellitus (T2DM) has substantially expanded the understanding of GLP-1 biology. These studies reveal broader effects on cellular stress responses, mitochondrial homeostasis, autophagy, and inflammatory pathways across multiple experimental systems [1,8,9,10,11]. Several GLP-1RAs have been shown in preclinical and clinical studies to cross the blood–brain barrier (BBB), although the extent of CNS penetration varies substantially between compounds and across species, with important implications for translational interpretation. In addition, GLP-1RAs engage conserved intracellular pathways, including cAMP-protein kinase A (PKA), phosphoinositide 3-kinase (PI3K)/Akt, and cAMP response element-binding protein (CREB) signalling, depending on the cellular context, all of which are strongly implicated in the regulation of neuronal survival, synaptic plasticity, and cellular resilience [1,12].

GLP-1R is expressed not only in peripheral tissues, including pancreatic β-cells and the gastrointestinal tract, but has also been reported in multiple brain regions associated with cognition, learning, and motor control, such as the striatum, midbrain, hindbrain, hypothalamus, and brainstem, in rodent and non-human primate models [13,14,15]. The activation of GLP-1R signalling engages a set of conserved intracellular pathways that operate across both central and peripheral tissues. In the periphery, GLP-1R activation elevates intracellular cAMP and engages PI3K/Akt and MAPK/ERK signalling to regulate insulin secretion, cell survival, and metabolic homeostasis [16,17,18]. These signalling cascades are also implicated in the regulation of apoptosis, mitochondrial biogenesis, autophagy, and inflammatory responses—processes central to neurodegenerative disease pathogenesis.

Consistent with this mechanistic overlap, the activation of central GLP-1R signalling in experimental models has been associated with reduced neuroinflammation and oxidative stress, modulation of the synaptic plasticity, and attenuation of the apoptotic pathways. In models of AD, GLP-1 has been reported to exert neuroprotective effects, in part, via cAMP/PKA-dependent mechanisms, while in PD models, GLP-1R activation has been linked to the preservation of dopaminergic neurons and reduced glial reactivity. Together, these findings suggest that GLP-1R signalling engages conserved molecular pathways, with potential relevance for modulating neurodegenerative processes across peripheral and central compartments.

Despite these promising findings, key questions remain regarding the molecular and cellular mechanisms through which GLP-1R signalling influences neurodegenerative processes. In particular, the relative contributions of central versus peripheral GLP-1 actions, the durability of GLP-1R-mediated neuroprotective effects, and the extent to which metabolic and inflammatory pathways intersect with canonical neurodegenerative mechanisms remain incompletely understood. Addressing these gaps is critical for evaluating the therapeutic potential of GLP-1RAs beyond their established metabolic indications.

The aim of this review is to synthesise current mechanistic, preclinical, and clinical evidence relating to GLP-1 and GLP-1R function in the context of PD. With particular emphasis on gut–brain axis interactions, conserved intracellular signalling pathways, and neuroprotective mechanisms, this article integrates insights from metabolic biology, neuroscience, and clinical research to clarify how GLP-1R signalling may influence neuronal survival, inflammation, and cellular homeostasis in PD and related neurodegenerative disorders. In doing so, the review highlights both the therapeutic potential of GLP-1RAs and the key unresolved questions that must be addressed to advance their translational development as potential disease-modifying interventions.

## 2. Molecular Mechanisms Underpinning GLP-1-Mediated Neuroprotection

**Figure 1:**
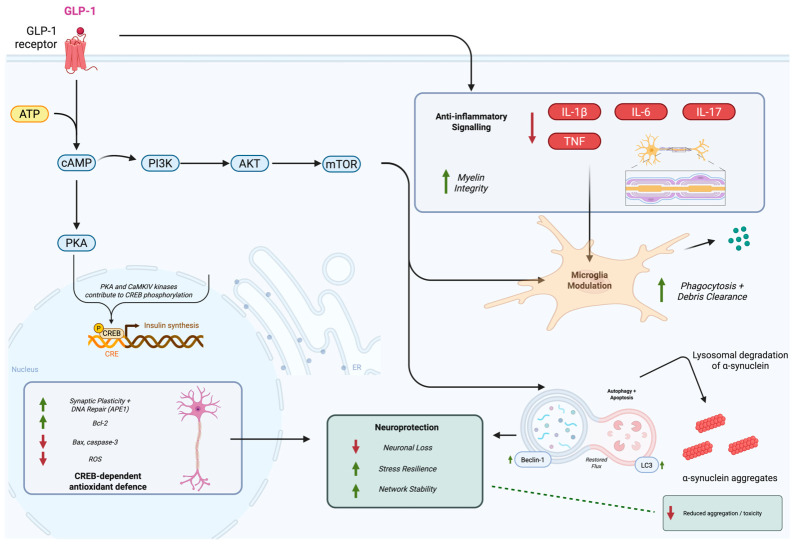
Molecular mechanisms underlying GLP-1-mediated neuroprotection.

Elucidating the molecular processes through which GLP-1 signalling confers neuroprotective effects is essential for informing therapeutic strategies in neurodegenerative disorders. Mechanistically, activation of the GLP-1R initiates intracellular signalling through heterotrimeric G proteins, with canonical Gαs-dependent stimulation of adenylate cyclase elevating intracellular cyclic adenosine monophosphate (cAMP) [16,17,18]. cAMP engages protein kinase A (PKA) and exchange protein directly activated by cAMP (EPAC), shaping transcriptional and functional programmes relevant to synaptic activity and cellular stress responses [19,20,21]. In parallel, GLP-1R activation recruits phosphatidylinositol-3-kinase (PI3K)/Akt signalling [22,23,24,25,26], a central regulator of survival and stress resilience. Downstream of these proximal nodes, GLP-1R signalling converges on conserved cascades, including mitogen-activated protein kinase/extracellular signal-regulated kinase (MAPK/ERK), Akt-dependent regulation of mammalian target of rapamycin (mTOR), glycogen synthase kinase-3β (GSK-3β), and Forkhead box O1 (FOXO1) [16]. Collectively, these pathways regulate neuronal viability, myelin integrity, inflammatory tone, oxidative stress, and cellular quality-control programmes, including autophagy and apoptosis. Together, they operate in a coordinated manner across neurons and glial cells, forming an integrated neuroprotective signalling network downstream of GLP-1R activation that enhances cellular resilience under pathological conditions (Figure 1).

GLP-1 receptor (GLP-1R) signalling engages conserved intracellular pathways in neurons and glial cells. Canonical Gαs-dependent signalling stimulates adenylate cyclase, increasing cAMP and activating PKA and CREB to promote transcription of pro-survival and antioxidant genes. In parallel, PI3K/Akt signalling supports neuronal survival, synaptic stability, and myelin-associated protein expression, while modulating mTOR activity to regulate autophagic flux and suppress apoptosis. GLP-1R signalling also attenuates oxidative stress and limits caspase-dependent cell death. In microglia, GLP-1R activation suppresses pro-inflammatory cytokine production and promotes a neuroprotective phenotype. In addition, GLP-1R activation enhances autophagy-lysosomal function, increasing key regulators such as Beclin-1 and LC3 to restore autophagic flux. Through this mechanism, GLP-1R signalling may facilitate the lysosomal degradation of both intracellular α-neuronal synuclein aggregates and pathogenic prion-like species released by neurons and taken up by neighbouring glia. Collectively, these coordinated effects support neuronal resilience under neurodegenerative and metabolic stress conditions. Arrows indicate directionality of effect (↑ increase; ↓ decrease), and dotted elements denote cellular debris undergoing clearance. Figure 1 was created in BioRender; Mezabrovschi, R. (2026).

### 2.1. Intracellular Signalling Pathways Activated by GLP-1 in Neurons

In neurons, GLP-1R signalling engages cAMP-dependent programmes that support neurotransmitter release and synaptic plasticity [27]. These canonical cAMP-PKA-CREB signalling events constitute a central pro-survival axis of GLP-1R activation in neurons (Figure 1, left panel).

GLP-1R activation has also been shown to protect cortical neurons from oxidative DNA damage via cAMP-dependent activation of the transcription factor CREB. CREB signalling induces the expression of apurinic/apyrimidinic endonuclease-1 (APE1), a key enzyme in the base excision repair (BER) pathway, thereby enhancing DNA repair capacity. Pharmacological inhibition studies suggest that APE1 induction depends on PI3K signalling rather than mitogen-activated protein kinase (MEK) activity in this experimental context. Consistent with this mechanism, administration of the GLP-1 analogue Exendin-4 (Ex4) enhances DNA repair in ischaemic stroke rat brains [28].

PI3K activation leads to phosphorylation of Akt, a critical mediator of neuronal survival. In vivo, activated Akt in the hippocampus is associated with protection against hypoxic stress and nitric oxide toxicity [29]. Akt signalling supports synapse formation and neuronal resilience and additionally promotes the expression of myelin-associated proteins, including myelin protein zero (MPZ) and peripheral myelin protein 22 (PMP22), linking GLP-1R signalling to the regulation of myelin-associated protein expression and Schwann cell function [30].

### 2.2. GLP-1-Mediated Myelin Protection: Peripheral and Central Mechanisms

In the peripheral nervous system (PNS), GLP-1RAs directly support myelinating glial cells. Ex4 enhances Schwann cell survival, proliferation, and migration and promotes neurite outgrowth in cultured dorsal root ganglion neurons. In immortalised adult rat Fischer Schwann cells (IFRS1), Ex4 upregulates the expression of MPZ and PMP22, two core structural components of the peripheral myelin sheath. These effects are mediated through activation of the PI3K/Akt pathway [31], linking GLP-1R signalling to glial differentiation and myelin maintenance in the PNS. Notably, this PI3K/Akt coupling also interfaces with mTOR-dependent control of autophagic flux (Figure 1), providing a mechanistic bridge between myelin maintenance and cellular quality control.

In contrast to the PNS, myelin preservation within the CNS appears to be mediated predominantly through indirect, immunomodulatory actions of GLP-1R signalling. Neuroinflammation is a major driver of oligodendrocyte dysfunction and demyelination in CNS disorders, including multiple sclerosis (MS). In experimental autoimmune encephalomyelitis and related MS models, Ex4 reduces demyelination, suppresses microglial activation, and improves clinical symptoms [32]. These effects are accompanied by marked reductions in pro-inflammatory cytokines, including IL-1β, IL-6, IL-17, and TNF-α, all of which are implicated in MS pathogenesis. Consistent with this mechanism, the long-acting GLP-1RA dulaglutide improves clinical outcomes in MS models by limiting lymphocyte infiltration into the CNS and suppressing encephalitogenic Th1/Th17 immune responses [33]. Together, these findings support a model in which CNS myelin protection by GLP-1R activation is mediated largely through the modulation of inflammatory/immune injury, although the direct effects on oligodendrocyte lineage cells remain less well defined across models.

In the context of PD, the preservation of myelin integrity may contribute to neuroprotection by maintaining axonal conduction, supporting metabolic coupling between neurons and glial cells, and limiting susceptibility to inflammatory and oxidative stress. Emerging evidence indicates that oligodendrocyte dysfunction and white matter abnormalities are present in PD, suggesting that myelin disruption may contribute to neuronal vulnerability. In this context, GLP-1R-mediated support of glial function and myelin integrity may indirectly preserve dopaminergic neuron health and network stability.

### 2.3. Regulation of Autophagy and Apoptosis in Neurons

Autophagy is a conserved lysosomal degradation pathway that maintains cellular integrity during stress. It removes damaged proteins and organelles to preserve energy balance and proteostasis. Its biological role is inherently context-dependent, acting as a cytoprotective stress response under adaptive conditions but contributing to regulated cell death when dysregulated [34].

The central control of autophagic flux is exerted by the PI3K/Akt/mTOR signalling axis, which integrates nutrient availability and growth factor cues. GLP-1RAs, particularly liraglutide, have been shown to engage this pathway in a disease- and context-specific manner. In models of neonatal hypoxic-ischaemic brain injury, liraglutide activates PI3K/Akt signalling, leading to the suppression of neuronal apoptosis and improved long-term cognitive performance, highlighting a mechanistic link between incretin signalling, autophagy regulation, and neuronal survival [35,36].

Although the primary focus of this section is neuronal autophagy and survival, it is notable that GLP-1R-mediated regulation of the PI3K/Akt/mTOR axis has been consistently observed across multiple metabolically stressed cell types, reinforcing the view that this pathway represents a conserved cytoprotective signalling axis. For example, Yao et al. demonstrated that liraglutide preserves nucleus pulposus cell viability in intervertrable discs during glucose toxicity through the restoration of PI3K/Akt/mTOR signalling, highlighting the robustness of this mechanism beyond the nervous system.

Similarly, in models of chemotherapy-induced injury, liraglutide attenuates oxidative damage and rescues PI3K/Akt/mTOR pathway homeostasis, resulting in reduced cellular apoptosis and improved functional outcomes. In the vascular compartment, diabetic oxidative stress activates reactive oxygen species (ROS)-dependent Akt and ERK1/2 signalling, driving excessive autophagy in endothelial cells while suppressing histone deacetylase-6 (HDAC6), thereby impairing cell migration and adhesion. GLP-1 signalling counterbalances this maladaptive response through coordinated engagement of the GLP-1R-ERK1/2-HDAC6 axis, reducing intracellular ROS, dampening pathological kinase activation, restoring HDAC6 expression, and normalising autophagic flux [37].

Importantly, analogous dysregulation of autophagy-lysosome function occurs in neurons and other highly energy-dependent cells. In pancreatic β-cells exposed to glucolipotoxic stress, the accumulation of autophagosomes, lysosomal impairment, and stalled autophagic flux culminate in cell death, a phenotype rescued by Ex4 through restoration of lysosomal activity and autophagic clearance [38]. While not neuronal per se, these findings provide mechanistic support for a convergent model in which GLP-1R activation stabilises autophagy-lysosome dynamics in cells with high metabolic demand, including metabolically vulnerable neuronal populations.

Apoptosis is a conserved, tightly regulated form of programmed cell death that eliminates irreversibly damaged cells and preserves tissue homeostasis. Although protective under physiological conditions, aberrant or sustained apoptotic signalling contributes to pathological cell loss. This is particularly relevant in post-mitotic neurons, where limited regenerative capacity amplifies the impact of apoptotic signalling.

Consistent with this framework, GLP-1R activation appears to co-ordinately suppress apoptotic signalling while enhancing autophagic clearance in neuronal systems. In models of spinal cord injury (SCI), Ex4 promotes autophagy while suppressing apoptosis, facilitating neuronal survival and regeneration. Higher Ex4 doses are associated with increased expression of LC3-II and Beclin1 and reduced caspase-3 levels, indicating enhanced autophagic activity [39]. Loss of Beclin1 in neurons leads to accumulation of amyloid-β peptides, whereas its overexpression reduces amyloid burden, highlighting a protective association between autophagy and AD pathology [40].

Mechanistically, stress-responsive Akt signalling can promote autophagy through stimulation of phosphatidylinositol-3-phosphate (PtdIns3P) production, facilitating autophagosome nucleation via recruitment of autophagy-related proteins. As a core component of the class III PI3K complex, Beclin1 is central to this process [41]. Concurrent suppression of caspase-3 further limits apoptotic cell death, enabling GLP-1R signalling to integrate lysosomal clearance pathways with pro-survival signalling in stressed neurons.

Importantly, both autophagy and apoptosis are inherently context-dependent processes, and their regulation must be tightly balanced to maintain cellular homeostasis. While GLP-1R activation has been widely associated with the promotion of adaptive autophagy and the suppression of excessive apoptosis, dysregulation of these pathways may have detrimental consequences. Excessive or prolonged autophagic activity can contribute to autophagy-associated cell death, particularly under conditions of sustained metabolic or oxidative stress. Conversely, persistent inhibition of apoptosis may allow the accumulation of damaged or dysfunctional cells, potentially exacerbating pathological processes. In this context, the effects of GLP-1R signalling on autophagy and apoptosis are likely to be highly dependent on disease stage, cellular environment, and the magnitude and duration of pathway activation. This highlights the importance of considering these pathways as dynamic and context-specific regulators of neuronal survival rather than uniformly protective mechanisms.

### 2.4. Modulation of Microglial Function

Microglia are the resident immune cells of the CNS and play essential roles in homeostasis and injury response. While transient activation supports repair, chronic microglial activation drives sustained neuroinflammation and neurodegeneration. GLP-1RAs modulate the microglial phenotype, promoting a shift from pro-inflammatory to anti-inflammatory states. In AD models, liraglutide reduces activated microglia in the hippocampus and cortex by roughly half in the reported experimental setting and decreases levels of IL-1β and TNF-α [42]. These effects highlight GLP-1RAs as modulators of neuroimmune homeostasis, acting not only on neurons but also on microglial inflammatory states (Figure 1, right panel).

Dual GLP-1/GIP receptor agonists exert enhanced anti-inflammatory effects by further suppressing microglial activation and reducing neurotoxic mediator release [43]. GLP-1RAs not only reduce inflammation but also increase the phagocytic ability of microglia, which facilitates the removal of amyloid-β and other pathogenic aggregates, hence increasing neuroprotection [44]. These results lend credence to the therapeutic potential of GLP-1RAs in neurodegenerative illnesses including Parkinson’s and Alzheimer’s, where inflammation caused by microglia is a major pathogenic factor [4].

### 2.5. GLP-1R Activation Promotes Neuronal Survival and Resistance to Oxidative Stress

By reducing oxidative stress and strengthening intrinsic resilience pathways, GLP-1R activation has been shown to confer robust neuroprotective effects in experimental systems. High levels of advanced glycation end products (AGEs) cause excessive production of ROS, leading to oxidative damage, mitochondrial dysfunction, and gradual neuronal death in both metabolic and neurodegenerative situations. Stressors like this are common in conditions like Parkinson’s and Alzheimer’s.

In PD-relevant settings, these stressors intersect with mitochondrial complex I dysfunction, dopamine-dependent redox cycling, and impaired proteostasis. By coordinating the activation of intracellular survival signalling cascades, GLP-1RAs can attenuate components of these stress responses in experimental systems. GLP-1R signalling engages PI3K/Akt-CREB pathways, promoting the transcription of pro-survival and antioxidant genes, which lowers intracellular oxidative stress and enhances neuronal metabolic performance [45].

In parallel, GLP-1R signalling simultaneously modifies the ratio of pro-apoptotic to anti-apoptotic pathways. It has been demonstrated that GLP-1RAs increase the expression of anti-apoptotic proteins like Bcl-2 while suppressing the expression of pro-apoptotic mediators like Bax and cleaved caspase-3. These effects result from the combined activation of the cAMP-PKA, PI3K/Akt, and CREB signalling pathways, which together restrict programmed cell death and promote neuronal survival [12].

Beyond preventing apoptosis, GLP-1R activation also supports neuronal repair and plasticity. By stabilising intracellular redox balance and sustaining survival signalling, GLP-1RAs facilitate neuronal differentiation and neurite outgrowth, processes essential for circuit maintenance and functional recovery following injury or neurodegeneration [46]. Together, these effects support the rationale for evaluating GLP-1RAs as candidate interventions for neurological conditions, including AD and PD, that are marked by oxidative stress-induced neuronal vulnerability and apoptotic cell death.

## 3. GLP-1R Signalling Architecture Across the Gut–Brain–Immune Axis

### 3.1. GLP-1 Receptor Signalling in the Central Nervous System

The CNS GLP-1 system contributes to endogenous regulation of glycaemia and energy homeostasis. Acute intracerebroventricular administration of the GLP-1R antagonist exendin-9-39 attenuates glucose utilisation and increases glycogen synthesis in skeletal muscle, supporting a contribution of central GLP-1R signalling to systemic glucose control [6]. Chronic pharmacological blockade of central GLP-1R or selective knockdown of GLP-1-producing neurons in the nucleus tractus solitarius (NTS) results in glucose intolerance, further highlighting the contribution of CNS GLP-1 pathways to metabolic homeostasis [47].

GLP-1R expression within the hypothalamus and brainstem—including the arcuate nucleus (ARC), paraventricular nucleus (PVN), ventromedial hypothalamic nucleus (VMH), and dorsal motor nucleus of the vagus (DMV)—underpins region-specific effects on insulin secretion, hepatic glucose production, and food intake [16,48]. GLP-1R activation in ARC neurons enhances β-cell insulin secretion and suppresses hepatic glucose output, while PVN GLP-1R signalling predominantly regulates appetite without significantly altering glycaemia. Additional forebrain control of glucose homeostasis may occur via polysynaptic parasympathetic pathways involving hypothalamic proopiomelanocortin (POMC) neurons projecting to the NTS and DMV [49].

GLP-1RAs are often described as exerting ‘central’ effects. However, their capacity for direct CNS engagement varies widely depending on molecular structure and pharmacokinetics. Short-acting peptide agonists such as exenatide and lixisenatide demonstrate limited but measurable BBB penetration in preclinical models, whereas long-acting agonists including liraglutide and semaglutide show minimal cerebrospinal fluid (CSF) exposure despite prolonged systemic half-lives [50,51,52,53,54]. Notably, rodents exhibit greater relative CNS uptake than humans [55], and preclinical doses frequently exceed human-equivalent exposures, necessitating caution when extrapolating mechanistic findings to human neurodegenerative disease [51].

Importantly, GLP-1RAs represent a pharmacologically heterogeneous class. Short-acting compounds generate transient receptor activation with relatively greater peak CNS exposure, whereas long-acting formulations provide sustained systemic signalling with reduced peak brain concentrations [56,57]. Extended-release platforms such as PT302 further modify CNS exposure profiles in rodent models [58]. These kinetic differences are likely to influence the balance between direct CNS actions and indirect peripheral-to-central signalling mechanisms.

Within the CNS, GLP-1R is widely distributed across the hypothalamic and brainstem nuclei involved in autonomic regulation, energy balance, and stress responses, as well as in regions implicated in neurodegeneration. GLP-1R-expressing cells are observed throughout the extent of the mouse brain, with high densities in the ARC, PVN, dorsomedial hypothalamic nucleus, and central amygdala [14,59]. Central GLP-1R signalling engages the conserved pathways outlined in Section 2, regulating mitochondrial function, autophagy, and inflammatory responses, supporting a mechanistic basis for its proposed relevance to PD and other neurodegenerative conditions.

### 3.2. Variations in GLP-1R Expression in the Brain Across Species: Consequences for Translation

Comparative studies reveal both conserved and species-specific features of GLP-1R distribution, with important implications for translation. Given that GLP-1 has a significant impact on feeding behaviour in rats, GLP-1R expression is prevalent in the hypothalamus of these species [60]. GLP-1R expression in the mouse brain is more regionally concentrated. For instance, the hindbrain nuclei and hypothalamus of the mouse brain show region-enriched GLP-1R expression [14,59]. Non-human primates exhibit significant hypothalamic expression as well [15], although their cortical regions differ noticeably. Recent high-resolution mapping of the human brain has demonstrated species-specific differences in GLP-1R distribution. Although hippocampal expression is conserved across mammals, in humans GLP-1R expression appears to be more prominent in the frontal cortex than in hypothalamic regions [61]. Furthermore, GLP-1R expression is minimal or absent in the human cerebellum and more widely distributed across cortical regions than reported in animal models, emphasising the need for caution when translating preclinical findings to human neurodegenerative disease. Available human brain datasets suggest a more diffuse cortical distribution of GLP-1R relative to rodents, with minimal regional specificity and no single dominant site (except from the hypothalamus). These cross-species differences in regional GLP-1R expression are summarised in Table 1. Reported regional differences in GLP-1R expression across species partly reflect methodological constraints, underscoring the need for multimodal validation when translating preclinical neuroprotective mechanisms to humans.

### 3.3. Peripheral GLP-1 Signalling and Vagus-Mediated Gut–Brain Communication

Because the intracellular signalling outputs of GLP-1R activation are discussed in detail in Section 2, this section focuses on where GLP-1R signalling is initiated across peripheral and central compartments and how incretin-derived signals are propagated between the gut, immune system, and brain.

While enteroendocrine L-cells represent the primary peripheral source, GLP-1 is also produced by preproglucagon-expressing neurons located in the NTS of the brainstem. CNS GLP-1 production enables local modulation of neural circuits, bypassing the rapid degradation of circulating GLP-1 by dipeptidyl peptidase-4 (DPP-4), which limits the half-life of peripheral GLP-1 to less than two minutes [63].

Peripheral GLP-1 influences central function through two principal routes: neural and humoral. The neural pathway, widely considered a major mechanism, involves GLP-1 acting on vagal afferent fibres in the gut or hepatic portal region, transmitting signals to the NTS. In contrast, the humoral pathway—the direct entry of GLP-1 into the brain across the BBB—is thought to play a more limited role due to rapid peripheral degradation [64]. Through these vagally mediated circuits, gut-derived metabolic signals are integrated into central autonomic and neuroendocrine control systems, providing a structural framework through which peripheral metabolic state can influence neuronal function and behaviour.

An important unresolved question is whether direct CNS engagement of GLP-1R is required for neuroprotective effects or whether peripheral mechanisms are sufficient to influence disease progression. While several GLP-1RAs demonstrate measurable, albeit limited, BBB penetration, the extent to which this contributes to clinical efficacy remains unclear. Notably, GLP-1R signalling in peripheral tissues can modulate systemic inflammation, metabolic homeostasis, and immune responses, all of which are increasingly recognised as contributors to neurodegenerative vulnerability. Through vagal afferent pathways and circulating mediators, these peripheral signals may influence central processes indirectly, including microglial activation, neuronal stress responses, and synaptic function. In this context, it is plausible that the therapeutic effects of GLP-1RAs arise from a combination of direct CNS actions and indirect systemic mechanisms, rather than requiring robust central receptor engagement alone. Resolving this distinction will be critical for optimising drug design, particularly in determining whether compounds should prioritise CNS penetrance or peripheral efficacy to achieve maximal neuroprotective benefits.

### 3.4. GLP-1R Distribution Across Peripheral Organs and Immune Interfaces

Beyond the gut–brain axis, GLP-1R is expressed across multiple peripheral tissues, positioning incretin signalling at key metabolic–immune interfaces. High levels of GLP-1R expression are observed in pancreatic β cells, where GLP-1 mediates glucose-dependent insulin secretion, suppresses glucagon release, delays gastric emptying, and reduces appetite [6,65]. Lower but functionally relevant expression has also been reported in the gastrointestinal tract, lungs, heart, kidneys, vasculature, pancreatic α cells, and the PNS [66,67].

These peripheral expression sites enable GLP-1 signalling to coordinate systemic metabolic responses while simultaneously modulating inflammatory tone and cellular stress resilience. Importantly, several of these tissues—including the gut epithelium and immune cell niches—represent interfaces through which peripheral inflammation and metabolic dysfunction may shape CNS vulnerability in PD.

Together with the central GLP-1R populations described above, these peripheral expression sites establish anatomical and functional continuity between metabolic, immune, and neural compartments. Through this distributed receptor architecture, GLP-1 signalling is positioned to integrate peripheral metabolic state, immune activation, and cellular stress responses, with CNS circuits implicated in vulnerability and resilience in PD. This organisation supports both direct central actions and indirect peripheral-to-central signalling routes, providing a structural framework through which incretin-based therapies may influence neurodegenerative processes under conditions of metabolic or inflammatory stress.

## 4. The Gut–Brain Axis in Parkinson’s Disease: Where Microbiota, Immunity, and GLP-1 Converge

### 4.1. Gut–Brain Axis Mechanisms Relevant to PD

Gastrointestinal dysfunction is a common and often early feature of PD [68,69], with symptoms such as constipation frequently preceding motor diagnosis by many years [70,71]. Reduced bowel frequency, delayed colonic transit, and other non-motor gastrointestinal disturbances are increasingly recognised as prodromal features in subsets of patients, although their specificity for PD remains limited. These observations support a role for the gut as a modifier of disease-relevant pathways rather than a primary causal site.

One mechanistic framework linking gastrointestinal dysfunction to PD-relevant pathology involves disruption of intestinal barrier integrity [72] and subsequent exposure to microbial products. The increased intestinal permeability reported in PD cohorts is proposed to permit translocation of bacterial components such as lipopolysaccharide (LPS) into the systemic circulation, driving chronic low-grade inflammation. In experimental systems, circulating endotoxin promotes microglial priming, lowering the threshold for exaggerated neuroinflammatory responses within the CNS [73]. In this context, systemic LPS exposure provides a mechanistic link between peripheral inflammation and central pathology by disrupting BBB integrity, enhancing cytokine trafficking into the CNS, and promoting sustained microglial activation. These processes create a pro-inflammatory neural environment that increases neuronal vulnerability, particularly within metabolically sensitive regions such as the substantia nigra. Accordingly, gut-derived inflammatory signals are best viewed as amplifiers of ongoing neurodegenerative processes rather than initiating events, providing a conceptual bridge to the microbiota-GLP-1-inflammation interactions discussed in Section 4.3 and Section 4.4.

In parallel, neural communication routes linking the gut and brain provide a pathway through which peripheral signals may influence central circuits relevant to PD. The vagus nerve, with projections to the nucleus NTS, integrates sensory input from the gastrointestinal tract and conveys information related to nutrient status, immune activation, and hormonal signalling. Altered vagal signalling has been proposed to modulate brainstem nuclei implicated in autonomic control and neuroinflammation [74], although the directionality and disease specificity of these effects remain incompletely defined. Within this framework, enteric nervous system pathology and α-synuclein accumulation observed in gastrointestinal tissues of some PD patients have been interpreted as consistent with gut–brain axis involvement [75], though it is recognised that such findings do not establish a unidirectional gut-origin model of disease.

GLP-1 signalling intersects with these gut–brain pathways at multiple levels. As an incretin hormone released from enteroendocrine L-cells, GLP-1 integrates nutrient sensing with vagal afferent signalling, immune modulation, and systemic metabolic control. GLP-1 and GLP-1RAs have been shown to influence inflammatory tone, cellular stress responses, and barrier function in peripheral tissues while also engaging the central neuroprotective pathways outlined in Section 2. These convergent properties position GLP-1 as a potential modulator of gut-derived inflammatory and neural signals that may impact CNS vulnerability in PD, providing a mechanistic rationale for examining microbiota–GLP-1 interactions in greater detail.

### 4.2. Microbiota Control of GLP-1 Secretion

Accumulating evidence indicates that microbial composition and metabolites critically shape GLP-1 synthesis, secretion, and downstream signalling. This microbiota–GLP-1 axis extends beyond glycaemic control and is increasingly implicated in neuro-metabolic and inflammatory pathways relevant to PD.

The disruption of gut microbial communities, including germ-free conditions or antibiotic exposure, impairs postprandial GLP-1 secretion across multiple experimental systems, highlighting a central role for microbiota in enteroendocrine function [76,77,78]. These effects are linked to altered bile acid regulation and nutrient sensing in the distal intestine, both critical for GLP-1 release. The restoration of microbial diversity, through faecal microbiota transplantation or bile acid supplementation, rescues GLP-1 secretion, underscoring the dependence of incretin signalling on an intact microbial ecosystem [79].

Specific microbial taxa (e.g., Lactobacillus [80], *Akkermansia muciniphila*) further modulate GLP-1 activity in a context-dependent manner [81], including antibiotic remodelling [82,83] and high-fat diet (HFD) models [84]. *Clostridium butyricum* reduces GLP-1-GLP-1R signalling in MPTP-treated mice despite improving dysbiosis, illustrating context-dependent effects in PD-relevant models [85,86].

Microbial metabolites act as key postbiotic regulators of GLP-1 release. Short-chain fatty acids (SCFAs) stimulate GLP-1 secretion via GPR41/43-dependent mechanisms [87,88,89]. Dietary modulation of SCFA availability enhances GLP-1 and PYY secretion from enteroendocrine cells [90,91] and dampens intestinal inflammation [92]. Additional metabolites, including L-tryptophan, promote GLP-1 production through transcriptional upregulation of *Gcg* and *Pcsk1* in L-cells and support β-cell regeneration in diabetic models [93]. In contrast, hydrogen sulphide produced by *Desulfovibrio* species suppresses GLP-1 expression, impairing host metabolic homeostasis [94].

Collectively, microbiota-derived signals exert bidirectional and context-dependent control over GLP-1 signalling. Beneficial taxa and metabolites enhance GLP-1 activity, whereas pathogenic species suppress it, supporting microbiota-GLP-1 interactions as a potential therapeutic target in PD and related disorders.

### 4.3. Bidirectional Interaction Between GLP-1 Signalling and the Gut Microbiota

There is growing evidence supporting bidirectional associations between the gut microbiota and GLP-1RA treatment [95,96]. Metagenomic analyses in T2DM patients show that GLP-1RAs alter microbial composition and function, reducing dysbiosis and improving metabolic balance [97]. In rodent models, liraglutide increases SCFA-producing taxa, including *Bacteroides*, *Lachnospiraceae*, and other advantageous probiotic species, and partially normalises the elevated Firmicutes-to-Bacteroidetes ratio observed in diabetes [96]. Liraglutide also improves renal outcomes in diabetic kidney disease models by increasing circulating L-5-oxoproline and modifying gut microbial composition, reducing lipid deposition in renal tubular cells and supporting a gut microbiota–metabolite–organ axis [98]. Together, these findings demonstrate that GLP-1RAs exert context-dependent effects on the gut microbiome, with implications extending beyond glycaemic control (Table 2).

GLP-1RAs also modulate gut microbial composition in metabolic disorders beyond T2DM, particularly the taxa linked to inflammation and glycolipid metabolism [105,106]. Moreover, liraglutide decreased *Proteobacteria*, which contains numerous pathogens, while increasing the abundance of *Firmicutes*, *Bacteroidetes*, and *Actinomycetes* in non-alcoholic fatty liver disease (NAFLD) patients [100]. In mice with NAFLD, liraglutide markedly increased the relative abundance of *Akkermansia*, *Romboutsia*, and selected members of the order *Bacteroidales* while significantly decreasing multiple taxa [99]. By mitigating HFD-induced gut dysbiosis, semaglutide exerted protective effects by increasing beneficial taxa like *Lachnospiraceae* and *Akkermansia* [101]. Semaglutide also greatly encourages the growth of *Blautia coccoides* and *Bacteroides acidifaciens*, which may aid in the synthesis of acetate [102].

However, the findings are not uniform. In a randomised controlled trial, combined liraglutide and sitagliptin treatment did not alter microbial composition or diversity in T2DM patients, although liraglutide was associated with increased deoxycholic acid (DCA), suggesting effects on bile acid metabolism [104]. Similarly, no significant microbiome changes were observed in older T2DM patients, aside from a non-significant increase in Alistipes, potentially influenced by small sample size and uncontrolled dietary factors [103]. Collectively, these findings indicate that GLP-1RAs exert context-dependent effects on the gut microbiome, with variability across disease states, treatments, and study designs, supporting bidirectional interactions between microbiota and GLP-1 signalling.

### 4.4. The Gut Microbiome, Inflammation, and GLP-1

There is increasing evidence of a bidirectional relationship between inflammation, gut microbiota, and GLP-1 signalling. Microbial dysbiosis can promote inflammatory responses and alter GLP-1 release, while GLP-1 itself can influence immune signalling and microbial composition. A key mediator of this interaction is LPS, a bacterial endotoxin that links dysbiosis to immune activation. Experimental studies in humans and animal models show that LPS can acutely stimulate GLP-1 secretion via Toll-like receptor-dependent mechanisms. For example, increased GLP-1 release during intestinal ischaemia supports a link between barrier dysfunction, endotoxin translocation, and incretin secretion under inflammatory stress [107,108].

Dysbiosis-associated loss of epithelial integrity further promotes LPS leakage into the circulation, driving chronic low-grade inflammation. In parallel, pro-inflammatory mediators, including IL-6, IL-1, and LPS, directly stimulate GLP-1 secretion from intestinal enteroendocrine L-cells, linking incretin release to immune activation. In metabolic disorders such as T2DM, this is accompanied by increased intestinal permeability and metabolic endotoxemia, characterised by elevated circulating LPS levels [109,110]. Consequently, altered microbial composition provides both a source of inflammatory stimuli and a permissive environment for sustained LPS exposure, creating conditions under which GLP-1 secretion and metabolic signalling may be aberrantly regulated. Importantly, these peripheral inflammatory signals are not confined to the gut but can propagate to the CNS, where LPS-induced cytokine release and microglial activation contribute to neuroinflammatory cascades implicated in PD pathogenesis. Through effects on BBB permeability and immune signalling, LPS-mediated inflammation provides a mechanistic pathway by which gut-derived immune activation may influence central neurodegenerative processes.

On the other hand, GLP-1RAs have shown positive immunomodulatory effects. GLP-1RA therapy changed the gut microbiome by boosting the number of advantageous species like *Lactobacillus* and *Bifidobacterium*, increasing anti-inflammatory cytokines IL-22 and IL-10 [111]. These findings support a feedback loop in which microbial metabolites regulate GLP-1 secretion, GLP-1 modulates immune and microbial homeostasis, and inflammation both drives and is shaped by dysbiosis.

### 4.5. GLP-1R Signalling in Peripheral Immune Cells: Conflicting Evidence and Open Questions

Beyond gut epithelial and microbiota-dependent mechanisms, GLP-1 signalling has been proposed to exert direct immunomodulatory effects on peripheral immune cells, potentially shaping systemic inflammation relevant to PD, although the underlying receptor dependence remains unresolved. Emerging evidence suggests that GLP-1 may exert immunomodulatory effects on peripheral immune cells, including T cells and macrophages, potentially influencing cytokine production and regulatory T cell differentiation [112]. Through effects on cytokine synthesis and cell proliferation, GLP-1 has been reported to modulate immune cell function within peripheral blood mononuclear cell (PBMC) populations, including effects on T cells and monocytes. GLP-1 has also been suggested to favour expansion of regulatory T cell subsets [113].

However, despite these reported effects, multiple studies indicate that GLP-1R expression in PBMCs is very low or undetectable. Several independent investigations report minimal GLP-1R expression in these cells, raising uncertainty as to whether observed immunomodulatory effects reflect direct receptor signalling or indirect, secondary mechanisms [114,115]. Resolving this uncertainty is particularly relevant in PD, where peripheral immune dysregulation and systemic inflammation are increasingly recognised as contributors to disease progression.

## 5. Brain Insulin Resistance and Metabolic Dysfunction in Parkinson’s Disease

### 5.1. Epidemiological Links Between Type 2 Diabetes and Parkinson’s Disease

Within this gut–immune–brain framework, insulin resistance provides a convergent molecular axis linking metabolic stress to the pro-survival and quality-control pathways engaged by GLP-1R signalling. Early evidence linking metabolic disease and PD was reported by Sandyk in 1993, who stated that individuals with PD and comorbid T2DM exhibited more severe motor symptoms and a reduced therapeutic response compared with non-diabetic PD patients [116]. Early clinical reports suggested a high prevalence of impaired glucose tolerance among individuals with PD, with estimates ranging from 50 to 80%; however, more recent population-based studies indicate that overt metabolic impairment is present in closer to 20% of cases [117].

Subsequent large-scale observational studies across multiple populations—including cohorts from the UK [118], Finland [119], Taiwan [120], the United States [121] and Denmark [122]—have reported an increased risk of PD among individuals with T2DM, with effect sizes varying substantially across cohorts (23–85%). Case-control studies further suggest that PD patients with T2DM may develop motor symptoms approximately one year earlier and exhibit greater disease severity compared with non-diabetic PD patients [123]. Neuroimaging studies have additionally revealed increased cortical atrophy in PD patients with T2DM, particularly within frontal regions associated with cognitive impairment [124,125].

Notably, several studies have reported an inverse association between T2DM and PD risk, underscoring the heterogeneity of observational findings and the influence of confounding factors [126,127], including medication exposure, vascular comorbidities, and survival bias.

### 5.2. Insulin Signalling in the Central Nervous System and Its Disruption in PD

Insulin exerts important functions within the CNS, extending beyond metabolic regulation to support neuronal survival, synaptic plasticity, mitochondrial function, and redox homeostasis, while restraining neuroinflammatory signalling. Accumulating evidence suggests that the PD brain exhibits features of insulin resistance, including impaired insulin receptor signalling and downstream pathway dysfunction [128]. Insulin receptors are highly expressed in the substantia nigra and basal ganglia—regions that undergo early and profound neurodegeneration in PD [129,130].

Disruption of insulin signalling in these regions has been proposed to contribute to PD pathogenesis through multiple convergent mechanisms, including impaired protein clearance, α-synuclein aggregation, lysosomal dysfunction, and mitochondrial stress [131]. These processes mirror pathogenic cascades observed in other neurodegenerative conditions linked to insulin resistance, reinforcing the concept of shared metabolic vulnerability across ageing-related brain disorders.

### 5.3. Shared Pathophysiological Pathways Linking Metabolic Dysfunction and Neurodegeneration

PD and T2DM are both age-associated disorders of increasing global prevalence and burden [132,133]. At the cellular level, PD and T2DM share several core pathological features. These include chronic inflammation, mitochondrial dysfunction, lysosomal impairment, and protein aggregation [130,134]. These overlapping mechanisms suggest that metabolic dysfunction may not merely coexist with PD but could influence disease susceptibility and progression.

Insights from AD further support this framework. AD has been described as exhibiting features of ‘type 3 diabetes’ due to strong links between brain insulin resistance, oxidative stress, tau hyperphosphorylation, dysregulated amyloid processing, and neuronal loss [135,136,137]. Although AD pathology differs from PD, there is a significant proportion of patients with co-pathology, and these findings underscore the broader principle that impaired insulin signalling within the brain promotes neurodegeneration through conserved molecular pathways. Importantly, insulin, insulin-like growth factor-1 (IGF-1), and GLP-1 share overlapping neuroprotective properties––including BBB penetration, regulation of synaptic plasticity, and activation of pro-survival signalling—positioning incretin pathways as attractive therapeutic targets across neurodegenerative contexts.

### 5.4. Metabolic Therapies as Modifiers of PD Risk and Progression

The interpretation of epidemiological links between T2DM and PD is complicated by medication effects. Several glucose-lowering therapies—including GLP-1RAs, metformin, and thiazolidinediones—have been associated with a reduced risk of PD onset in observational studies [138,139,140,141]. These associations raise the possibility that the modulation of insulin and incretin signalling pathways may influence neurodegenerative risk, potentially obscuring underlying disease relationships in population-based analyses.

Additional confounders, including elevated body mass index, vascular risk factors, and hypoglycaemic episodes, further complicate causal inference [119,142,143]. Hypoglycaemia itself may contribute to neurodegeneration through neuroglycopenia-induced neuronal injury, contributing to both vascular and non-vascular cognitive decline.

Notably, the interpretation of these associations is complicated by the potential confounding effects of pharmacological interventions. Glucose-lowering therapies, including GLP-1RAs, metformin, and thiazolidinediones, are known to modulate key pathways implicated in neurodegeneration, including inflammation, mitochondrial function, oxidative stress, and autophagy. As a result, observed associations between T2DM and PD risk or progression may reflect, at least in part, the biological effects of these treatments rather than the underlying metabolic disorder itself. Distinguishing between disease-driven and treatment-mediated effects remains a significant challenge and underscores the need for carefully controlled biomarker-informed studies to disentangle these contributions.

## 6. Neuroprotective Effects of GLP-1 Receptor Agonists in Preclinical Models

A substantial body of preclinical work implicates GLP-1R signalling in neuroprotective responses across multiple experimental systems. Although many studies originate from AD models, the underlying mechanisms—including mitigation of oxidative stress, modulation of autophagy and apoptosis, preservation of synaptic integrity, and regulation of glial reactivity [9]—are highly relevant to PD, where similar cellular stress pathways are implicated in dopaminergic neuron vulnerability.

### 6.1. Cell-Based Models: Neuronal Survival and Differentiation

At the cellular level, GLP-1RAs exert direct neuroprotective effects in neuronal systems. In SH-SY5Y human neuroblastoma cells, liraglutide pretreatment significantly attenuated oxidative stress while promoting neuronal survival and differentiation-related phenotypes, primarily by activating the PI3K-Akt and Akt-STAT3 signalling pathways [144,145]. These pathways play key roles in maintaining neuronal structure, preventing apoptosis, and maintaining mitochondrial integrity—processes that are compromised in PD. Similar findings were reported across independent SH-SY5Y studies [146], supporting the reproducibility of GLP-1-associated neuroprotective signalling in neuronal cells, which underpins the functional recovery observed in animal models.

### 6.2. Mouse Models

GLP-1RAs have been extensively tested in transgenic mouse models of AD, but the signalling mechanisms identified are also highly relevant to PD, particularly where they intersect with mitochondrial dysfunction, glial activation, and impaired protein clearance. Liraglutide continuously decreased tau hyperphosphorylation, amyloid burden, and neuroinflammatory markers in APP/PS1, APP/PS1xdb/db, 5xFAD, and triple-transgenic APP/PS1/Tau models [147,148,149,150]. It also increased autophagic flux, maintained synapses, restored insulin signalling, and improved neurovascular integrity. These effects were observed following systemic, intranasal, and cell-based delivery, suggesting effective central engagement of GLP-1R-associated pathways.

Preclinical studies further demonstrate that GLP-1RAs attenuate cognitive impairment in AD models by reducing pathogenic hallmarks such as amyloid plaque burden and oligomeric amyloid species [5,151]. In parallel, GLP-1RAs improve memory-related behaviours and suppress microglial activation, highlighting an immunomodulatory component of GLP-1R signalling [152]. Notably, the long-acting GLP-1RA NLY01 potently suppresses pro-inflammatory microglial activation and limits astrocytic conversion to neurotoxic reactive phenotypes, thereby protecting hippocampal neurons from hypoxia- and glutamate-induced excitotoxicity [153]. This glia-centred mechanism is particularly relevant to PD, where neuroinflammation and non-cell-autonomous glial toxicity are increasingly recognised as important contributors to dopaminergic neuron degeneration.

Beyond amyloid-centred effects, dulaglutide was demonstrated to effectively penetrate the BBB and enhance learning and memory in intracerebroventricular-streptozotocin (STZ)-induced AD animal models via activating the PI3K/Akt/GSK-3β pathway [154]. Exenatide treatment restored normal systemic glucose metabolism and decreased brain amyloid-β levels in 3xTg-AD mice with STZ-induced diabetes [155], suggesting a link between metabolic regulation and central neuroprotective effects.

Moreover, exenatide enhanced cognitive function and decreased astrocytic oxidative stress and neuroinflammation in 5xFAD mice, with evidence consistent with inhibition of NLRP2 inflammasome activation [156]. Beyond amyloid-centred effects, GLP-1RAs influence intracellular signalling cascades linked to neurodegeneration as well as synaptic function. Through enhanced production of GluR1 and postsynaptic density protein-95 (PSD-95), Ex4 has been demonstrated to improve CREB phosphorylation and raise brain-derived neurotrophic factor (BDNF) levels, enhancing synaptic plasticity and neurite integrity [157] alongside increased ADAM10-mediated α-secretase activity. Such effects are particularly relevant to PD, where early synaptic dysfunction precedes overt dopaminergic neuron loss. Collectively, these in vitro data support a role for GLP-1R activation in modulating neuronal survival and differentiation-associated pathways relevant to neurodegeneration. These molecular and cellular effects are accompanied by improvements in behavioural outcomes, including enhanced learning and memory and, in PD-relevant models, the preservation of motor function. The concordance between pathway modulation and functional endpoints supports the translational relevance of GLP-1R signalling in neurodegenerative disease models.

Exenatide has shown neuroprotective effects in dopaminergic systems, which is significant for PD. Treatment with a sustained-release version of exenatide (PT320) increased dopaminergic signalling within midbrain circuits and improved motor function in MitoPark mice [158], supporting a role for GLP-1RAs in preserving nigrostriatal function in this model.

In APP/PS1/Tau mice, lixisenatide, a brain-penetrant GLP-1RA, decreased tau pathology, amyloid deposition, and microglial activation; these effects were associated with increased PKA/CREB signalling and the suppression of p38/MAPK pathways [159]. Notably, in an MPTP mouse model of PD, lixisenatide similarly enhanced motor outcomes, supporting the potential relevance of GLP-1R signalling to dopaminergic neurodegeneration [160].

### 6.3. Rat Models

Rat models provide complementary insight into the cellular mechanisms underlying GLP-1-mediated neuroprotection. In intracerebroventricular-STZ-induced rat models and non-human primates infused with amyloid-β oligomers, liraglutide improved memory performance, supporting partially conserved effects across species.

Ex4 has been particularly well characterised in rat systems. Ex4 restored cAMP and phosphorylated CREB levels, normalised intracellular calcium dynamics, and reversed Aβ1-42-induced deficiencies in long-term potentiation in hippocampal CA1 areas, highlighting its function in calcium homeostasis and synaptic integrity [161]. In rat models of intracerebroventricular-STZ, Ex4 attenuated memory decline, decreased neuronal death, increased cell division, and stimulated synaptogenesis in the hippocampus [162].

Further studies demonstrated that Ex4 improved cognitive performance, reduced amyloid-β accumulation, restored acetylcholine levels, and improved mitochondrial function through PI3K/Akt-dependent pathways [163]. Additional neuroprotective effects included reduced brain TNF-α levels, preservation of choline acetyltransferase activity, and protection against hippocampal neuron loss [164]. Ex4 also downregulated GSK-3β activity, reversing tau hyperphosphorylation, and safeguarded neurons from degeneration under metabolic stress [165]. Collectively, these rat studies indicate that GLP-1RAs influence synaptic plasticity, mitochondrial function, inflammatory signalling, and calcium homeostasis—processes that are also disrupted in PD. Thus, these molecular effects are accompanied by measurable improvements in cognitive performance, synaptic function, and neuronal survival in vivo, indicating that modulation of these pathways translates into functional benefits.

However, important limitations of these model systems must be considered when interpreting their translational relevance. Many preclinical studies rely on acute toxin-induced paradigms, such as the MPTP mouse model or intracerebroventricular STZ rat models, which induce rapid and severe neuronal injury. While these systems are valuable for interrogating specific molecular pathways, they do not fully recapitulate the slow, progressive, and age-dependent nature of PD in humans. They lack key features of pathology and disease chronicity, including gradual dopaminergic neuron loss, long-term compensatory adaptations, and the complex interplay between genetic susceptibility, metabolic dysfunction, and environmental factors. Consequently, the therapeutic effects observed in these models may overestimate neuroprotective efficacy when translated to clinical settings. These limitations underscore the need for complementary approaches, including genetic and ageing-based models, as well as careful interpretation of preclinical findings when assessing their relevance to human disease. Taken together, while a substantial proportion of the preclinical literature derives from AD models, the mechanisms highlighted here have been discussed specifically in relation to PD because they converge on pathogenic pathways central to dopaminergic neuron vulnerability and disease progression.

## 7. Clinical Evaluation of GLP-1 Receptor Agonists in Neurodegenerative Diseases

Encouraged by accumulating preclinical evidence for neuroprotection, several GLP-1RAs approved for the treatment of T2DM—including exenatide, liraglutide, semaglutide, and lixisenatide—have been advanced into clinical investigation for neurodegenerative disorders. The translational relevance of these experimental findings has been examined through multiple randomised controlled trials (RCTs) and observational studies. To date, clinical evaluation has focused predominantly on PD and AD, offering insight into safety, tolerability, and potential disease-stage dependence of GLP-1R-based therapeutic strategies in the human brain. In addition to being FDA-approved or undergoing trials for the treatment of T2DM, the GLP-1RAs exenatide (Bydureon^®^, Byetta^®^, PT320, NLY01), liraglutide (Victoza^®^), semaglutide (Ozempic^®^, Rybelsus^®^), and lixisenatide (Adlyxin^®^/Lyxumia^®^) are also being investigated for potential utility in neurodegenerative diseases.

### 7.1. Clinical Evidence in Parkinson’s Disease

Several RCTs have investigated whether GLP-1RAs can modify neurological outcomes in individuals living with PD. Early clinical evidence emerged from a 2013 single-blind proof-of-concept trial involving 45 patients with moderate PD, in which 12 months of exenatide treatment was associated with significant improvements in the Movement Disorders Society Unified Parkinson’s Disease Rating Scale (MDS-UPDRS) compared with non-use [166]. These findings provided the early clinical evidence that GLP-1R activation may influence disease-relevant motor outcomes.

Subsequently, a randomised, placebo-controlled trial in 62 patients with moderate PD demonstrated that exenatide treatment attenuated deterioration in the MDS-UPDRS part III off-medication motor score relative to placebos [167]. Importantly, motor benefits persisted beyond the treatment period. This raises the possibility that effects extend beyond symptomatic improvement. Supporting a potential class effect, a recent placebo-controlled trial reported that lixisenatide mitigated progression in MDS-UPDRS part III scores compared with placebos [168].

Liraglutide has also been evaluated in PD populations. A 52-week Phase II trial of liraglutide demonstrated that treatment was safe and well tolerated in PD patients and was associated with improvements in non-motor symptoms, mobility, and overall quality of life [169]. More recently, a 14-month double-blind Phase II trial in early-stage PD reported a modest but statistically significant improvement in motor outcomes, supporting the hypothesis that earlier intervention may be associated with improved outcomes [168]. Notably, this trial, conducted in patients diagnosed within three years of symptom onset, highlights disease stage as a potential determinant of therapeutic responsiveness to GLP-1R-based interventions.

However, not all trials have demonstrated positive outcomes. The EXENATIDE-PD3 trial, a large Phase III placebo-controlled study, followed 194 PD patients over 96 weeks and did not identify a significant difference in MDS-UPDRS part III scores between exenatide and placebo groups [170]. This discrepancy with earlier trials may reflect, among other factors, differences in disease stage, treatment duration, outcome sensitivity, or underlying biological heterogeneity within PD populations across trials. Consistent with this variability, a Cochrane systematic review concluded that there is currently “low certainty” evidence supporting improvement in motor impairment in PD patients treated with GLP-1RAs [171].

Mechanistic insight into clinical effects has been strengthened by biomarker analyses. In PD patients treated with exenatide, neuronal-derived exosomes isolated from blood samples showed alterations in insulin-, Akt-, and mTOR-related signalling pathways, consistent with mechanisms identified in preclinical dopaminergic models [172]. These findings support the notion that GLP-1RAs engage disease-relevant intracellular signalling cascades within neurons in vivo. Together, these data suggest engagement of central GLP-1-associated pathways beyond metabolic control, aligning with experimental evidence consistent with the preservation of dopaminergic neurons and suppression of neurotoxic astrocytic phenotypes in PD models [9,173,174].

Collectively, these findings highlight considerable variability in clinical outcomes across GLP-1RA trials in PD. While early-phase studies suggested potential symptomatic or disease-modifying effects, larger and more recent trials have produced mixed or negative results. This inconsistency indicates that the clinical efficacy of GLP-1RAs is not yet established and is likely influenced by factors including disease stage, compound-specific properties, and trial design. These observations emphasise the need for a more critical evaluation of clinical data and are explored further in Section 7.4.

### 7.2. Real-World Evidence and PD Risk Modification

In parallel with interventional trials, large-scale real-world observational studies have examined whether exposure to GLP-1RAs is associated with modification of PD risk. Leveraging multinational electronic health record data, a retrospective cohort study of individuals with T2DM without prior neurodegenerative disease compared new users of GLP-1RAs with those initiating dipeptidyl peptidase-4 inhibitors (DPP-4i) or basal insulin [175]. Overall, initiation of GLP-1RAs was associated with a lower incidence of diagnosed neurodegenerative disease compared with DPP-4 inhibitors or basal insulin.

When analyses were restricted specifically to PD incidence, no significant difference in the risk of new-onset PD was observed between GLP-1RA and DPP-4 inhibitor initiators; however, sensitivity analyses demonstrated an approximately 15% lower risk of PD onset among GLP-1RA users compared with those initiating basal insulin therapy. Although observational in nature, these findings are consistent with the biological plausibility of GLP-1R-mediated neuroprotection and provide a rationale for rigorously designed clinical trials to evaluate the disease-modifying potential of GLP-1RAs in neurodegenerative disorders, potentially in populations stratified for genotype, phenotype or specific biochemical biomarkers.

Interpretation of these findings regarding GLP-1RAs requires caution. DPP-4 inhibitors themselves may confer biochemical and clinical benefits relevant to PD outcomes [176,177], potentially attenuating detectable differences between treatment groups. In addition, PD diagnosis in this study relied on ICD-10 coding, which has been reported to misclassify Parkinsonian disorders in approximately 50% of cases [178,179], limiting diagnostic specificity and potentially obscuring true associations.

### 7.3. Clinical Evidence in Alzheimer’s Disease

Although not the primary focus of this review, clinical trials in AD provide valuable mechanistic context for GLP-1R signalling in the human brain, particularly where they inform target engagement, metabolic effects, and pathways shared with PD. Several placebo-controlled RCTs have examined liraglutide in patients with mild-to-moderate AD dementia. A 2016 study involving 38 participants demonstrated that 26 weeks of liraglutide treatment prevented decline in cerebral glucose consumption, although no significant effects on cognitive performance were observed [180]. Functional MRI studies further demonstrated increased brain connectivity following liraglutide treatment, although cognitive measures remained largely unchanged [181]. Additional PET studies reported attenuation of further decline in cerebral glucose uptake in AD patients without diabetes, again in the absence of clear cognitive benefit [182].

A larger multicentre Phase IIb trial (NCT1843075) involving 204 patients did not demonstrate a significant effect on the primary endpoint of cortical glucose metabolism [183]; however, liraglutide was found to be well tolerated, and there were numerical trends across secondary outcome measures that comprise different markers of AD and neuroinflammation, such as changes in brain volume and connectivity, tau phosphorylation, amyloid-β levels, microglial activation, and several cognitive measures [184].

Exenatide has also been evaluated in early AD, where its safety and potential central effects were assessed in an 18-month Phase II randomised controlled trial. Treatment was well tolerated and was associated with reduced amyloid-β42 levels in neuronal-derived extracellular vesicles, consistent with target engagement within the CNS [185]. However, no significant differences were observed between exenatide and placebo groups with respect to cognitive performance, cortical thickness or volume, or established AD biomarkers measured in CSF or plasma. The absence of clear clinical or structural benefit likely reflects limited statistical power and highlights the challenges of detecting durable clinical effects in early-stage AD within relatively small cohorts. Although AD pathology differs from PD, these studies are mechanistically informative, as many of the same processes—mitochondrial dysfunction, impaired autophagy, oxidative stress, and glial activation—also contribute to dopaminergic neuron degeneration in PD. To facilitate comparison across compounds evaluated in metabolic and neurodegenerative settings, the following table (Table 3) summarises key GLP-1RAs, highlighting differences in molecular structure, half-life, receptor affinity, BBB penetration, and current clinical indications.

### 7.4. Implications for PD-Focused Therapeutic Development

Collectively, clinical studies indicate that GLP-1RAs are generally safe and well tolerated in neurodegenerative disease populations. While results in AD have been mixed and largely confined to metabolic and imaging endpoints, clinical evidence in PD—particularly with exenatide, liraglutide, and lixisenatide—suggests potential benefits in motor and non-motor domains in selected cohorts. The divergence between early-phase positive trials and later large-scale studies underscores the importance of disease stage, trial design, biomarker integration, and patient stratification.

Despite strong preclinical evidence, a clear translational gap exists between experimental findings and clinical outcomes in PD. This discrepancy likely reflects a combination of biological, pharmacological, and methodological factors that influence the effectiveness of GLP-1RAs in human populations. Recent negative or inconclusive clinical trial outcomes, including those observed with NLY01 and PT302, highlight important challenges in translating preclinical GLP-1R biology into clinical efficacy. Several non-mutually exclusive factors may contribute to these discrepancies. First, insufficient central target engagement remains a key consideration. Several GLP-1RAs exhibit limited or variable BBB penetration in humans despite evidence of CNS activity in preclinical models. Second, pharmacokinetic and pharmacodynamic differences between compounds—including receptor bias, duration of receptor activation, and tissue distribution—may influence the extent and nature of downstream signalling in the human brain. Third, variability in trial design, including differences in endpoint sensitivity, treatment duration, and patient selection, may limit the ability to detect disease-modifying effects. Enrolment of patients at relatively advanced disease stages may reduce the capacity to observe neuroprotective benefits, which are likely to be most effective earlier in the disease course. Finally, species-specific differences in GLP-1R expression, CNS exposure, and disease biology complicate direct extrapolation from preclinical models to human PD.

An additional key consideration for future trial design is the identification of patient subgroups most likely to benefit from GLP-1R-based therapies. Given the established links between metabolic dysfunction and neurodegeneration, individuals with comorbid insulin resistance or T2DM may represent a particularly responsive population, as GLP-1R signalling directly targets pathways implicated in both metabolic and neuronal stress. Similarly, patients characterised by elevated systemic or central inflammatory markers may derive greater benefit from the immunomodulatory effects of GLP-1RAs. Disease stage is also likely to be critical, with earlier intervention potentially offering greater neuroprotective benefit before substantial dopaminergic neuron loss has occurred. In addition, stratification based on age at onset or underlying genetic and molecular profiles may further refine patient selection.

Future PD trials will benefit from incorporating molecular and cellular biomarkers of GLP-1R engagement to support patient stratification and assessment of target engagement. Several approaches are emerging, although many remain at an early stage of validation. Among these, neuronal-derived extracellular vesicles isolated from peripheral blood offer a minimally invasive means of interrogating intracellular signalling pathways, including insulin-, Akt-, and mTOR-related processes. While initial studies suggest that these measures may be responsive to GLP-1RA treatment, further work is required to establish their robustness, specificity, and utility in clinical trial settings. In parallel, the development of neuroimaging strategies, including PET tracers targeting GLP-1R or downstream metabolic pathways, may offer direct insight into central target engagement, although these approaches remain in early stages of development. Peripheral biomarkers, including circulating inflammatory mediators (e.g., TNF-α, IL-6) and metabolic markers, may further complement these approaches by capturing systemic responses to GLP-1R activation.

Together, improved assessment of CNS target engagement, careful patient stratification and biomarker-driven trial designs are needed to evaluate the therapeutic potential of GLP-1RAs in neurodegenerative disease.

## 8. Conclusions

Accumulating evidence positions GLP-1R signalling in a mechanistic nexus linking metabolic dysfunction to neurodegenerative vulnerability. Across experimental systems, GLP-1R activation engages conserved intracellular pathways—including cAMP/PKA, PI3K/Akt, CREB, and mTOR—that regulate neuronal survival, mitochondrial function, autophagy-lysosome dynamics, and inflammatory responses. These processes are central to PD pathogenesis, where dopaminergic neurons exhibit heightened sensitivity to metabolic stress, impaired proteostasis, and chronic neuroinflammation.

Epidemiological associations between T2DM and PD, together with evidence of brain insulin resistance independent of peripheral metabolic status, provide a biological framework that contextualises GLP-1R signalling as a disease-relevant pathway rather than a purely metabolic target. Within this framework, GLP-1RAs function as modulators of stress-response networks shared across peripheral and central tissues. Preclinical studies consistently demonstrate that GLP-1R activation mitigates oxidative injury, stabilises mitochondrial and lysosomal homeostasis, modulates microglial reactivity, and preserves synaptic and dopaminergic integrity.

Clinical translation has yielded heterogeneous outcomes, with early-phase trials in PD suggesting potential benefits, while larger studies highlight the influence of disease stage, pharmacokinetics, and biological heterogeneity. Together, these findings indicate that GLP-1R signalling represents a coherent mechanistic axis in PD, warranting continued investigation using biomarker-guided trial designs capable of resolving disease-modifying effects.

## Figures and Tables

**Table 1:** Comparative distribution and detection of GLP-1R expression across species.

Species	Brain Regions with Reported GLP-1R Expression	Relative Distribution Pattern	Primary Detection Method(s)	Key Translational Considerations	Representative References
Mouse	Hypothalamus (ARC, PVN, DMH), hindbrain nuclei (NTS, AP), central amygdala	Regionally enriched; high hypothalamic and brainstem concentration	Transgenic reporter mice (GLP-1R-Cre), RNAscope in situ hybridisation, autoradiography	Strong hypothalamic bias may overestimate metabolic circuit engagement relative to humans	[14,59]
Rat	Hypothalamus, brainstem, select midbrain regions	Prominent hypothalamic expression	Radioligand binding, in situ hybridisation	Feeding-related effects prominent; cortical expression less well defined	[60,62]
Non-human Primates	Hypothalamus, hippocampus, limited cortical regions	Intermediate between rodent and human	In situ hybridisation, immunohistochemistry	Improved translational relevance for CNS engagement versus rodents	[15]
Human	Frontal cortex, hippocampus; minimal cerebellar expression	More diffuse cortical distribution; less regional dominance	Single-nucleus RNA-seq, bulk RNA-seq, autoradiography	Cortical bias may favour cognitive over metabolic effects; limits direct rodent extrapolation	[61]

**Table 2:** Gut microbiota alterations associated with GLP-1RA treatment across experimental and clinical contexts.

GLP-1RA	Model/Disease Context	Microbial Changes (↑ Increase/↓ Decrease)	Associated Metabolic or Inflammatory Effects	Primary Detection Method	Key References
Liraglutide	T2DM (human, rodent)	↑ SCFA-producing taxa (*Bacteroides*, *Lachnospiraceae*); ↓ *Firmicutes/Bacteroidetes* ratio; ↓ *Proteobacteria*	Improved glycaemic control; reduced metabolic inflammation	16S rRNA sequencing; metagenomics	[95,96]
Liraglutide	NAFLD (human, mouse)	↑ *Akkermansia*, *Romboutsia*, *Bacteroidales*; ↓ *Klebsiella*, *Anaerotruncus*	Reduced hepatic steatosis; decreased pro-inflammatory cytokines	16S rRNA sequencing	[99,100]
Liraglutide	Diabetic kidney disease (rodent)	Altered microbial composition linked to ↑ circulating 5-oxoproline	Reduced ectopic lipid deposition; renal protection	Metabolomics + 16S sequencing	[98]
Semaglutide	High-fat diet (mouse)	↑ *Lachnospiraceae*, *Ruminococcus*, *Akkermansia*; ↑ *Blautia coccoides*, *Bacteroides acidifaciens*	Reduced dysbiosis; inverse correlation with TNF-α, IL-1β, IL-6	16S rRNA sequencing	[101,102]
Liraglutide + Sitagliptin	T2DM (human)	No significant change in α- or β-diversity; ↑ deoxycholic acid; ↑ *Alistipes* (NS)	Potential bile-acid-mediated metabolic effects	Shotgun metagenomics; bile acid profiling	[103,104]

**Table 3:** Pharmacological properties of GLP-1 receptor agonists and clinical trial outcomes in Parkinson’s disease.

GLP-1RA	Type	Half-Life	Receptor Affinity	Clinical Use	PD Trial Results	Notes
Exenatide	Synthetic exendin-4	~2.4 h	Very High	T2DM, PD trials	Slowed motor decline in PD [167,186]	Derived from Gila monster saliva; short-acting
Exenatide ER	Synthetic exendin-4 (extended-release)	~5 days	High	T2DM, PD trials	Phase 3 PD trial in progress (EXENatide-PD3)	Weekly injection, sustained effect
Lixisenatide	Synthetic exendin-4 derivative	~3 h	Moderate	T2DM	Led to a slower progression of motor disability [168]	Short-acting, daily injection
Liraglutide	Human GLP-1 analogue (fatty acid-linked)	~13 h	Moderate	T2DM, obesity, PD trials	Mixed results, some neuroprotective signals, but small sample size [187]	Daily dosing, increased albumin binding
Semaglutide	Human GLP-1 analogue (modified)	~7 days	High	T2DM, obesity, PD trials	PD trials in early stages (NCT03659682)	Weekly injection or oral formulation
Dulaglutide	GLP-1 fused to IgG-Fc	~4 days	High	T2DM	Not yet tested in PD	Reduced renal clearance
Tirzepatide	Dual GLP-1/GIP agonist	~5 days	Very High	T2DM, obesity	No PD trials yet	Enhances both GLP-1 and GIP signalling
NLY01	Pegylated GLP-1RA	~12 days	High	PD trials	Ineffective in early PD trials [188]	Designed for neuroprotection; reduces glial activation
PT302	Sustained-release exenatide (microsphere formulation)	~2 weeks (functional release)	High	PD trials	Phase 2 PD trial completed; did not meet primary efficacy endpoint but demonstrated acceptable safety and target engagement	Long-acting formulation designed to improve CNS exposure and adherence; monthly injection

## Data Availability

No new data were generated or analysed in this study. This article is a review of previously published work, and all relevant data are included within the cited references.

## Abbreviations

The following abbreviations are used in this manuscript:

GLP-1	Glucagon-Like Peptide 1
GLP-1R	Glucagon-Like Peptide 1 Receptor
GLP-1RAs	Glucagon-Like Peptide 1 Receptor Agonists
GPCR	G-protein-coupled receptors
GIP	Glucose-dependent insulinotropic polypeptide
PD	Parkinson’s Disease
AD	Alzheimer’s Disease
T2DM	Type 2 diabetes mellitus
BBB	Blood–brain barrier
CNS	Central Nervous System
PNS	Peripheral nervous system
cAMP	Cyclic Adenosine Monophosphate
CREB	cAMP response element-binding protein
PKA	Protein Kinase A
PI3K	Phosphoinositide 3-Kinase
AKT	Protein Kinase B
ERK	Extracellular Signal-Regulated Kinase
MAPK	Mitogen-activated protein kinases
EPAC	Exchange protein activated by cAMP
mTOR	Mammalian target of rapamycin
GSK-3β	Glycogen synthase kinase-3β
FOXO1	Forkhead box O1
DNA	Deoxyribonucleic acid
RNA	Ribonucleic acid
APE1	Apurinic/Apyrimidinic endonuclease 1
BER	Base excision repair
Ex4	Exendin-4
MPZ	Myelin protein zero
PMP22	Peripheral myelin protein 22
IFRS1	Immortalised Fischer rat Schwann cells
MS	Multiple sclerosis
IL-1β	Interleukin-1 beta
IL-6	Interleukin-6
IL-17	Interleukin-17
TNF-α	Tumour necrosis factor-alpha
Th1	T helper type 1
Th17	T helper type 17
ROS	Reactive oxygen species
HDAC6	Histone Deacetylase 6
LC3	Microtubule-associated protein 1A/1B-light chain 3
SCI	Spinal cord injury
PtdIns3P	Phosphatidylinositol-3-phosphate
AGEs	Advanced glycation end-products
NTS	Nucleus tractus solitarius
ARC	Arcuate nucleus
PVN	Paraventricular nucleus
VMH	Ventromedial hypothalamic nucleus
DMV	Dorsal motor nucleus of the vagus
POMC	Proopiomelanocortin
CSF	Cerebrospinal fluid
DPP-4	Dipeptidyl peptidase-4
DPP-4i	Dipeptidyl peptidase-4 inhibitors
PYY	Peptide YY
LPS	Lipopolysaccharide
HFD	High-fat diet
MPTP	1-methyl-4-phenyl-1,2,3,6-tetrahydropyridine
SCFAs	Short chain fatty acids
GPR41/43	G protein-coupled receptor 41/43
DKD	Diabetic kidney disease
NAFLD	Non-alcoholic fatty liver disease
DCA	Deoxycholic acid
PBMC	Peripheral blood mononuclear cell
IGF-1	Insulin-like growth factor-1
STAT3	Signal transducer and activator of transcription
APP	Amyloid Precursor Protein
PS1	Presenilin 1
5xFAD	Five familial Alzheimer’s Disease mutation
STZ	Streptozotocin
NLRP2	NLR family pyrin domain containing 2
GluR1	Glutamate receptor 1
PSD-95	Postsynaptic density protein-95
BDNF	Brain-derived neurotrophic factor
ADAM10	A disintegrin and metalloproteinase domain-containing protein 10
RCTs	Randomised controlled trials
PET	Positron Emission Tomography
